# Associations between markers of health and playing golf in an Australian population

**DOI:** 10.1136/bmjsem-2019-000517

**Published:** 2019-04-12

**Authors:** Brad Stenner, Amber D Mosewich, Jonathan D Buckley, Elizabeth S Buckley

**Affiliations:** 1 Alliance for Research in Exercise, Nutrition and Activity (ARENA), University of South Australia, Adelaide, South Australia, Australia; 2 Faculty of Kinesiology, Sport, and Recreation, University of Alberta, Edmonton, Alberta, Canada; 3 University of South Australia Cancer Research Institute, University of South Australia, Adelaide, South Australia, Australia

**Keywords:** golf, well-being, exercise, sport

## Abstract

**Objective:**

To investigate associations between markers of health and playing golf in an Australian population.

**Methods:**

Secondary analysis of data from the Australian National Nutrition and Physical Activity Survey to compare selected health outcomes between golfers (n=128) and non-golfers (n=4999).

**Results:**

Golfers were older than non-golfers (mean±SD 57.7±14.2 years, 48.5±17.6 years, p<0.05). A higher proportion of golfers were overweight or obese compared with non-golfers (76% vs 64%, p<0.05), and golfers were more likely to have been diagnosed with ischaemic heart disease (IHD) at some time in their life (OR 2.8, 95% CI 1.0 to 7.8). However, neither the risk of being overweight or obese (OR 1.4, 95% CI 0.9 to 2.2) or having been diagnosed with IHD (OR 2.1, 95% CI 0.8 to 5.8), were significant after controlling for age. Golfers were more physically active than non-golfers (8870±3810 steps/day vs 7320±3640 steps/day, p<0.05) and more likely to report high health-related quality of life (HRQoL) than non-golfers (OR 1.8; 95% CI 1.0 to 3.3), but not after adjusting for physical activity (OR 1.4, 95% CI 0.9 to 2.2).

**Conclusion:**

Compared with non-golfers, golfers were more likely to be overweight or obese and to have been diagnosed with IHD, but not after adjusting for golfers being older. Golfers were more likely to report a higher HRQoL, but not after adjusting for golfers being more physically active. There may be an association between golfers being more physically active than non-golfers and reporting a higher HRQoL.

**What is known**

- Golf is a source of moderate intensity exercise and can be played throughout the lifespan.
- Golfers experience improved longevity compared with non-golfers, and golf can contribute to improved cardiovascular health and overall physical health.

**Significant new findings**

- We report a positive association between golf and quality of life/well-being.

## Introduction

Golf is played in more than 200 countries and territories around the world and by ~60 million people.^1^ In Australia and the USA, ~5% and ~8% of the population play golf, respectively.^2–4^ In England, golf is also popular, with ~2% of the population playing regularly and is consistently ranked in the top five sports for participation in both England and Australia.^3 5^

Regular moderate to vigorous physical activity benefits physical and mental health and decreases the burden of disease.^6–8^ A recent review found that, regardless of whether assessed using metabolic equivalents, heart rate or oxygen uptake, golf was primarily a moderate intensity physical activity.^9^ Consequently, golf participation could provide a range of health benefits for those who play regularly. A recent scoping review of the health benefits of golf participation identified 49 studies reporting positive associations between regular golf participation and improved life expectancy and physical health.^10^ Three studies^11–13^ showed a reduction in cardiovascular risk factors in healthy participants who played golf regularly, and two high-quality randomised control trials suggested that golf may improve cardiorespiratory fitness and assist with recovery from cardiac events.^12–14^ The walking involved in playing golf contributes to daily step count, which is widely recognised to not only be associated with improved physical health^8 10 13 14 16^ but also with improved mental health and well-being.^17–19^ Murray *et al* identified 29 studies that evaluated the associations between playing golf regularly and mental health and well-being, but no consistent effect on mental health was found. However, there was some evidence that golf positively impacted general mood, self-identity and social connectedness in healthy participants^20^ and those with severe mental health illness.^21^ Lane and Jarrett^22^ found that golf was associated with worsened mood following play; however, these immediate post-performance measures may have been due to poor performance during the round of golf rather than an effect of overall participation in the game itself. There are no data on the effects of golf participation on long-term well-being and health-related quality of life (HRQoL), other than indirect evidence indicating that participation in moderate to vigorous physical activity, which would include golf, can improve HRQoL.^17 18^

Thus, while there is good evidence for cardiovascular health benefits of regular golf participation, there is conflicting evidence around benefits for mental health and little information about effects on HRQoL. The purpose of the present study was to explore associations between golf and health, including HRQoL, in an Australian population. In 2011–2012, the Australian Bureau of Statistics (ABS) undertook a national survey of a stratified random sample of Australians which evaluated physical activity and sport participation, including golf, and selected health parameters. The data from that survey provided an opportunity to evaluate associations between golf participation and health.

## Methods

The Australian National Nutrition and Physical Activity Survey of 2011–2012 formed one component of a broader Australian Health Survey. The Physical Activity component of the National Nutrition and Physical Activity Survey collected data relating to the type of physical activity/sport completed by participants in the previous 7 days, along with self-reported and/or objective measures of health and well-being. Data were obtained via computer assisted face-to-face interview and either a home based or clinic visit to collect blood samples and other objective health measures. (For further information refer to ABS, Australian Health Survey 2011–2012 Explanatory Notes, http://www.abs.gov.au/australianhealthsurvey).

The National Nutrition and Physical Activity Survey was conducted from May 2011 to June 2012. Data were collected from ~9500 private dwellings throughout Australia. Initially, each state and territory was divided into geographically homogenous areas using the Australian Standard Geographical Classification, and then further divided into collection areas. Households in each collection area were randomly selected, providing a stratified random sample drawn from all states and territories (except for very remote areas) and as such is representative of the Australian population (refer ABS, Australia Health Survey 2011–2012 Users Guide, http://www.abs.gov.au/australianhealthsurvey). In each selected household, general demographic information was collected on all persons, and detailed information was collected from one adult and one child aged 2–17 years (ABS, 2013). A total of 12 153 persons participated in the survey. Of these, 9435 were aged 18+ and had demographic data relevant to the analysis available. However, as not all demographic and medical data were able to be linked with physical activity data, the sample size reduced for a number of the outcomes of interest, as indicated in table 1.

**Table 1:** Descriptive data

Characteristic	Non-golfers	Golfers	Total
	Mean (SD)	Mean (SD)	Mean (SD)
Age (years) (non-golf n=9307, golf n=128)	48.5 (17.6)	57.7* (14.2)	48.7 (17.6)
Income $A/week (non-golf n=8378, golf n=114)	1641.40 (1480.40)	1824.00 (1437.77)	1643.90 (1479.95)
Average steps/day (non-golf n=4999, golf n=76)	7314.6 (3635.6)	8865.7* (3806.4)	7337.8 (3642.7)
Total cholesterol mmol/L (non-golf n=3714, golf n=54)	5.15 (1.03)	5.39 (1.1)	5.15 (1.03)
HDL cholesterol mmol/L (non-golf n=3714, golf n=54)	1.37 (0.37)	1.43 (0.43)	1.37 (0.37)
LDL cholesterol mmol/L (non-golf n=3091, golf n=50)	3.17 (0.90)	3.40 (0.93)	3.17 (0.90)
HbA1c mmol/mol (non-golf n=3703, golf n=54)	36.64 (7.25)	36.38 (4.38)	36.64 (7.22)
Fasting glucose, mmol/L (non-golf n=3129, golf n=50)	5.2 (0.94)	5.3 (0.7)	5.2 (0.94)
BMI kg/m^2^ (non-golf n=7846, golf n=112)	27.5 (5.58)	28.4(4.39)	27.5 (5.56)
Proportion overweight or obese (BMI >25)	63.9%	75.9%*	64.1%

*Significantly different from non-golfers (p<0.05).

BMI, body mass index; HDL, high-density lipoprotein; HbA1c, glycated haemoglobin; LDL, low-density lipoprotein.

For the present analysis, demographic data reporting sex, age, birthplace, employment and income were extracted from the database, along with data for physical activity and sedentary behaviour (type, duration and intensity), medical conditions that had lasted, or were expected to last for 6 months or more, and physical measurements and biomedical markers of disease.

The International Classification of Disability mini classification was used to classify reported medical conditions and data were dichotomised into either ‘ever had’ or ‘never had’ the specified medical condition. HRQoL was measured using question 1 of the 12-Item Short Form Health Survey (SF-12)^23^ questionnaire which asked, ‘*In general, would you say your health is: excellent, very good, good, fair or poor?*’. Self-report measures for types of physical activity were used, and pedometers and daily activity sheets were used to collect objective physical activity data.

Blood and/or urine samples were collected from a subsample of participants (~3700) who also volunteered to provide biomedical data, and blood samples for analysis of total cholesterol, low-density lipoprotein, HbA1c and fasting glucose were also collected. Further detailed information regarding the sample, methodology and results can be obtained from the ABS (abs.gov.au/ausstats).

Categorical and continuous data related to outcomes of interest (ie, medical conditions and risk factors) were dichotomised into normal/abnormal levels or ‘has’/’has not had’ condition. Continuous data for daily step count were averaged for analysis. Data on type of physical activity were used to identify individuals as either having had participated in golf in the previous 7 days or not. For this analysis, it was assumed that persons who had participated in golf in the previous 7 days were regular golfers.

Individual (de-identified) data were accessed remotely via a secure link to the ABS database. Stata V.14 was used for all data management and statistical analysis. Descriptive statistics were calculated, and golfers and non-golfers were compared using Student’s t-tests for normally distributed variables and χ^2^ test for categorical variables. To explore associations between golf and medical and health markers, logistic regression was used to calculate ORs, including 95% CI, with and without adjusting for important confounders (physical activity, income as proxy for socioeconomic status and age). Statistical significance was set at p<0.05 for all analyses.

## Results

### Demographic data

Table 1 shows demographic characteristics for golfers and non-golfers. Golfers were older than non-golfers and more physically active. There was no difference in income between golfers and non-golfers, which was used as a proxy for socioeconomic status. While, overall, there was no difference detected in average body mass index (BMI) between golfers and non-golfers, a significantly higher proportion of golfers were overweight or obese compared with non-golfers. There were no other significant demographic differences between the two groups.

### Association between golf participation and markers of health

In relation to objective measures of health and well-being, compared with non-golfers, golfers had 83% higher odds of reporting high HRQoL in the unadjusted model (table 2). After adjusting for age, the odds of reporting a high quality of life were even higher for golfers (2.31×); however, after adjusting for physical activity levels, the odds of reporting a higher HRQoL was no longer significantly different between golfers and non-golfers.

**Table 2:** Association between golf participation and selected medical conditions and risk markers

Medical condition or risk marker	Unadjusted OR (95% CI)	OR adjusted for age (95% CI)	OR adjusted for physical activity (95% CI)	OR adjusted for physical activity and age (95% CI)
High cholesterol (total) (non-golf n=3714, golf n=54)	1.48 (0.86 to 2.54)	1.29 (0.75 to 2.22)	1.42 (0.78 to 2.58)	1.20 (0.66 to 2.20)
High LDL (non-golf n=3091, golf n=50)	1.28 (0.73 to 2.26)	1.14 (0.65 to 2.03)	1.13 (0.60 to 2.15)	0.99 (0.52 to 1.89)
Glycaemic control (HbA1c) (non-golf n=3703, golf n=54)	0.35 (0.05 to 2.53)	0.24 (0.03 to 1.76)	0.59 (0.08 to 4.37)	0.42 (0.06 to 3.10)
Elevated plasma glucose (non-golf n=3129, golf n=50)	0.89 (0.27 to 2.90)	0.64 (0.19 to 2.10)	1.49 (0.44 to 5.00)	1.11 (0.33 to 3.73)
Overweight or obese (BMI >25) (non-golf n=7846, golf n=112)	1.77* (1.15 to 2.75)	1.44 (0.93 to 2.24)	1.87* (1.07 to 3.25)	1.41 (0.81 to 2.47)
High blood pressure (>140/90) (non-golf n=7979, golf n=110)	1.30 (0.86 to 1.98)	0.92 (0.59 to 1.42)	1.33 (0.78 to 2.27)	0.83 (0.48 to 1.43)
High quality of life (SF-12) score (non-golf n=9307, golf n=128)	1.83* (1.03 to 3.26)	2.31* (1.30 to 4.13)	1.27 (0.57 to 2.80)	1.50 (0.68 to 3.32)
Condition: self-reported (ever diagnosed) (non golf n=9307, golf n=128)				
Diabetes (type II)	0.81 (0.26 to 2.57)	0.60 (0.19 to 1.91)	1.67 (0.52 to 5.42)	1.06 (0.33 to 3.47)
Elevated blood glucose	2.12 (0.92 to 4.87)	2.04 (0.89 to 4.71)	2.05 (0.74 to 5.71)	1.97 (0.70 to 5.52)
High cholesterol (total or LDL)	2.57* (1.61 to 4.10)	2.04* (1.27 to 3.28)	2.29* (1.22 to 4.30)	1.61 (0.86 to 3.06)
Hypertension	1.00 (0.58 to 1.72)	0.73 (0.42 to 1.27)	1.09 (0.54 to 2.22)	0.70 (0.34 to 1.44)
Ischaemic heart disease	2.83* (1.02 to 7.80)	2.06 (0.74 to 5.76)	4.15* (1.26 to 13.69)	2.27 (0.67 to 7.68)
Tachycardia	1.48 (0.60 to 3.66)	1.21 (0.49 to 2.99)	1.56 (0.48 to 5.04)	1.27 (0.39 to 4.12)
Disease of the arteries	3.12 (0.97 to 10.05)	2.30 (0.71 to 7.45)	4.57* (1.07 to 19.59)	2.95 (0.68 to 12.82)
Low blood pressure	0.37 (0.05 to 2.69)	0.43 (0.06 to 3.11)	0.59 (0.08 to 4.29)	0.75 (0.10 to 5.47)
Kidney disease	1.19 (0.29 to 4.88)	1.03 (0.25 to 4.23)	1.01 (0.14 to 7.41)	0.92 (0.13 to 6.78)

*Significant OR for golfers compared with non-golfers at p<0.05.

BMI, body mass index; HbA1c, glycated haemoglobin; LDL, low-density lipoprotein; SF-12, 12-Item Short Form Health Survey.

There was no difference in the odds of having high cholesterol between golfers and non-golfers; however, the odds of ever having had high cholesterol or being diagnosed with ischaemic heart disease (IHD) was significantly higher for golfers compared with non-golfers. The odds of having been diagnosed at some point in their life with either of these conditions was higher after adjusting for physical activity, but was no longer different between golfers and non-golfers after controlling for age. Similarly, golfers had 77% higher odds of being overweight or obese (ie, BMI >25 kg/m^2^), but this was no longer significantly different from non-golfers after adjusting for age.

There were no differences detected between golfers and non-golfers for other medical conditions or risk markers.

## Discussion

### New findings and explanations

This study provides new insights into the health and well-being of golfers. Despite being older, more likely to be overweight/obese and more likely to have had high cholesterol or been diagnosed with IHD, golfers were more physically active than non-golfers and more likely to report higher HRQoL. While these new data point to possible associations between golf and health, as the data were cross-sectional, we cannot comment on causality and, because of the relatively low numbers of golfers in the study, we were likely underpowered to detect differences in a number of the outcomes of interest. Adequately powered randomised controlled trials are required to test whether golf participation improves health.

Our finding that golfers were more likely to report higher HRQoL compared with non-golfers may have been due to golfers being more physically active than non-golfers, because there was no longer a significant difference between the two groups after controlling for physical activity. On average golfers performed ~1500 steps per day more (~21%) than non-golfers which, over 1 week, would equate to ~10 500 steps, which is roughly equivalent to the number of steps taken during one round of golf per week.^9^ The importance of physical activity to overall quality of life has been extensively described in the literature 6–8 17–19 25 and the results from this study suggest that the extra physical activity obtained by playing golf may contribute to improved HRQoL, despite higher odds of golfers having a range of medical conditions that might impact adversely on quality of life.

Moderate to vigorous intensity physical activity protects against the development of a range of chronic diseases.^24 25^ While data from the present study indicated that golfers were more physically active than non-golfers, because pedometry was used, it was not possible to determine the intensity of the physical activity. However, if the increased physical activity was due to playing golf, which is classified as moderate intensity exercise,^26^ then the additional physical activity might provide protection against development of a number of chronic conditions, including cardiovascular disease, type 2 diabetes and some cancers, as well as overweight and obesity. In Australia, almost 66% of Australians aged over 18 years are overweight or obese,^27^ which is consistent with the 64% of people who were overweight or obese in the present study. Data from the present analysis indicated that golfers were more likely to be overweight or obese than non-golfers, but after adjusting for age, there was no longer a difference. This suggests that the older age of golfers contributed to the higher odds of being overweight or obese compared with non-golfers, rather than anything directly related to playing golf.

Golfers were more likely than the non-golfers to have been diagnosed with high cholesterol and ischaemic heart disease at some time in their lives. Given that high cholesterol is a risk factor for developing IHD, it is perhaps not surprising that higher odds of having been diagnosed with elevated cholesterol was also associated with higher odds of having been diagnosed with IHD. However, there was no difference in total cholesterol levels measured in blood samples collected at the time of the study (refer table 1). The odds of having been diagnosed at some time with high cholesterol for golfers compared with non-golfers were reduced, but still significantly higher, after adjusting for age or physical activity, but was no longer significant after adjusting for both (refer table 2). The longer a person lives, the greater the chance is that they will at some time be diagnosed with hypercholesterolaemia; the Australian Health Survey indicated that the prevalence of dyslipidaemia (any abnormal blood lipid measure including cholesterol) increases with age, to a peak prevalence of 81% in people aged 65–74.^27^ Accordingly, after controlling for age, the odds of golfers having high cholesterol compared with non-golfers was reduced from 1.48 to 1.29.

The odds of golfers having been diagnosed with IHD was higher than non-golfers, and this was still higher after controlling for their higher level of physical activity, but not after controlling for age. The prevalence of IHD increases with age^27^ and therefore, because the golfers were older, this may have accounted for their elevated risk of having been diagnosed with IHD. The results of our analysis should be interpreted with some caution, given the cross-sectional nature of the data, but are consistent with a previous controlled trial that demonstrated the potential of golf to reduce risk factors associated with IHD.^11^

Our data showed that golfers were more physically active than non-golfers and that this may have contributed to their reporting a higher HRQoL. There was no significant difference between golfers and non-golfers in prevalence and/or relative odds of common medical conditions, such as high blood pressure, total cholesterol and diabetes. This suggests that golfers may have been managing their medical issues better than non-golfers, or that golf was being used as a means to increase physical activity to manage these conditions. Alternatively, golfers may have had fewer medical conditions that were not measured in the survey on which this study was based.

### Strengths and limitations

The National Nutrition and Physical Activity Survey provided an opportunity to examine associations between golf participation and measures of health status using a stratified random sample that is likely to be representative of the Australian population. Three per cent of the sample reported playing golf, which is consistent with known participation rates,^3 28^ suggesting that the sample is likely to be reflective of golf participation within the broader community. However, as the data were cross-sectional, the associations between golf participation and markers of health status that were identified cannot be interpreted as being causative.

In addition, while this study was large and representative of the Australia population, it included only 128 golfers. It is likely, given the small number of golfers, that the study may have been underpowered to detect significant differences in some variables. This is an important consideration, particularly in relation to key demographic variables such as income, as a measure of socioeconomic status. Socioeconomic status is one of the biggest determinants of overall health and could explain differences in health markers between our populations. We did not adjust for income in our analysis as the 11% higher average weekly income of golfers was not significantly different from that of non-golfers. Future research should endeavour to more closely evaluate the impact of socioeconomic status on HRQoL in golfers. Furthermore, an α level of 0.05 was used throughout to denote statistical significance with no adjustment for multiple comparisons. However, the study was exploratory and hypothesis generating in nature, and the associations found should be evaluated in subsequent studies to determine the impact of golf on health status.

There were also some key aspects of the data that should be noted. First, some measures were based on self-report, while others were objective measures, and self-report measures are subject to recall bias. The self-reported measures in the data set related to having ever been diagnosed with a medical condition, irrespective of time of diagnosis or follow-up treatment/management that may have assisted or perhaps resolved the medical conditions. The HRQoL measure was self-report and used one question only, rather than the full set of available items in the SF-8, SF-12 or SF-36, which would provide information on which domains of life (ie, physical functioning, role limitations, pain, general health, vitality/energy, social function and mental health) were responsible for the overall higher HRQoL in golfers. Therefore, in the present study, this question has been interpreted as reflecting HRQoL rather than overall quality of life. In addition, important data related to the length of time of playing golf and frequency of participation was not available, so further associations between how often golf is played and possible links to health remain unknown.

The nature of the data collected meant that not all data that may influence the outcome of interest could be utilised and included in the analysis. In addition, the Australian Health Survey consists of two parts: (1) data related to health and medical status and (2) data related to nutrition, physical activity and some medical status items, but not all participants complete both parts of the survey. Therefore, it was not possible to link all relevant data for each participant. Medication, diet and detailed physical activity (other than step count) were not available for the golfing participants, and as these are known to influence health biomarkers and outcomes, the effect of these is unknown for this population.

### Implications for the golfing industry and health professionals

Evidence for golf as a sporting activity that contributes to the health and well-being of its participants is developing. Previous work has identified golf as a source of appropriate physical activity for many, including older adults and those with cardiovascular disease, and that it can contribute to the overall health and well-being of people who experience reduced mental health. However, there is a lack of high-quality evidence in this area and further, well-designed studies are required. As a sport that can be played from very young to well into our older years, golf can contribute to health and well-being across the lifespan, and promotional messaging related to health and well-being could be considered as part of future recruitment campaigns to increase golf participation. The notion of using golf as an addition to exercise programmes is worthy of consideration. Golf is not likely to solve the problem of low levels of physical activity worldwide, but playing golf might be considered as a viable and appropriate adjunct to other strategies to increase physical activity to achieve medical and/or health benefits.

## Conclusion

This is the first study to use a large, representative data set of the Australian population to explore associations between playing golf and health. We report an association between playing golf and higher levels of HRQoL. Golfers are more physically active than the non-golfing population, and this may be contributing to their perceived higher HRQoL, despite them being older, and more overweight/obese. These results not only support possible health benefits of golf but also highlight the need for future studies to further explore links between golf and health. Future studies should, where possible, focus on establishing causative relationships between golf and health and include not only physical health but also mental health.
